# The Relationship between Health Literacy, Quality of Life, and Subjective Health: Results of a Cross-Sectional Study in a Rural Region in Germany

**DOI:** 10.3390/ijerph17051683

**Published:** 2020-03-05

**Authors:** Anna T. Ehmann, Oliver Groene, Monika A. Rieger, Achim Siegel

**Affiliations:** 1 Institute of Occupational and Social Medicine and Health Services Research, University Hospital Tübingen, Wilhelmstraße 27, 72074 Tübingen, Germany; anna.ehmann@med.uni-tuebingen.de (A.T.E.); monika.rieger@med.uni-tuebingen.de (M.A.R.); 2 OptiMedis AG, Burchardstraße 17, 20095 Hamburg, Germany; o.groene@optimedis.de; 3 London School of Hygiene and Tropical Medicine, University of London, London WC1E 7HT, UK

**Keywords:** health literacy, HLS-EU-Q16, quality of life, health status, integrated healthcare systems

## Abstract

Low health literacy is associated with poorer health and quality of life. An open question is whether a regional integrated healthcare system whose management strives to enable and empower its members with regard to health issues can contribute to a higher level of health literacy in the population. Against this background, in a cross-sectional study we surveyed a random selection of members of an integrated healthcare system in southwestern Germany (*n* = 180). The standardized questionnaire included, inter alia, questions on health literacy and subjective health. In this sample we wanted to (1) determine the proportion of respondents with sufficient health literacy and (2) investigate whether the association between health literacy and quality of life and subjective health status—as found in other studies—can be replicated. In our sample a health literacy score could be calculated in 126 subjects (70%). A sufficient level of health literacy was detected in 62% of respondents. Confirming the findings of a meta-analysis based on international studies, we found moderate correlations between health literacy and quality of life (r = 0.41) and health literacy and subjective health status (r = 0.40); these correlations hardly decreased when we controlled for various sociodemographic characteristics. As the proportion of respondents with sufficient health literacy was higher in our sample than in comparable studies conducted in Germany, we may hypothesize that an integrated healthcare system like the one we surveyed could have contributed to increased health literacy in the population. Thus, it could be worthwhile to investigate this research question with a more rigorous study design and a larger sample.

## 1. Introduction

According to a common definition, health literacy “entails people’s knowledge, motivation and competences to access, understand, appraise, and apply health information in order to make judgments and take decisions in everyday life concerning healthcare, disease prevention and health promotion to maintain or improve quality of life during the life course” [1].

In the course of the two preceding decades, health literacy has definitively become a high priority in health care. Compared to the Anglo-American region, Germany has taken up the debate and public attention on health literacy rather late [2,3]. The European Health Literacy Survey (HLS-EU), started in 2009 [4], made this topic increasingly important in Germany [2]: according to a more recent representative study, approximately every second adult in Germany has limited health literacy [5], i.e., every second adult has difficulties in accessing, understanding, appraising, and applying health-related information. This makes it difficult for them to make decisions in daily life that benefit their health [6]. Insufficient health literacy obviously harms those affected and may have negative effects on societies [7,8]. Individuals’ health literacy is closely—and positively—associated with their social, economic, and health status. If health literacy is limited, this has a negative effect on the individual’s state of health and their health behavior [9]. Low health literacy is linked to poorer health and quality of life. According to a recent meta-analysis, there is a moderate correlation (r = 0.35) between health literacy and quality of life across several international studies [10]. However, there are big differences between the studies, and in various studies various methodologies are used to capture health literacy [10]. Similarly, previous research findings differ in terms of the influence of factors such as age, educational level, and gender on the relationship between health literacy and health status [11].

The WHO’s ‘Shanghai Declaration on promoting health in the 2030 Agenda for Sustainable Development’ postulates that ‘health literacy empowers and drives equity’ [12]. Taking up this impulse of the WHO for Germany, the ‘National Action Plan Health Literacy’ (NAP) [6] was introduced in 2018. In fostering health literacy in Germany, the reduction of health inequity is a central objective of the NAP: the potentials of health literacy promotion should be used to offer equal health opportunities to differing groups [6].

In our article we focus on health literacy in a specific setting and population: our study population consists of members of a regional integrated healthcare system whose leading actors aim, among other things, to empower and activate their members so that these are enabled to manage their health issues and patient–provider interactions more competently. The members of the integrated healthcare system are at the same time members of two statutory health insurance funds AOK Baden-Württemberg (AOK BW) or SVLFG (Sozialversicherung für Landwirtschaft, Forsten und Gartenbau—the statutory health insurer for employees in agriculture, forestry, and horticulture). Both health insurers traditionally cover social strata and groups that are rather educationally disadvantaged, i.e., groups that tend to have less favorable preconditions for acquiring high levels of health literacy. Thus, the setting and study population provide an interesting case because we investigate a study population in which we would actually expect a below-average level of health literacy; at the same time, however, this population undergoes a population-based intervention aimed at empowering and activating the population with regard to health issues—which could lead to increased health literacy, too. The integrated healthcare system is located in the Kinzig valley in the Black Forest in southwestern Germany and is called ‘Integrierte Versorgung Gesundes Kinzigtal’ (‘Healthy Kinzig Valley Integrated Care’—‘GK’. It is one of the few population-based integrated healthcare systems in Germany, covering all sectors and indications of the population living there [13,14], and is often considered as a best-practice example for integrated healthcare in Germany [15]. At the time of the survey, about 70,000 people were living in the region. Almost half of them—about 33,000 persons—were insured by either AOK BW or SVLFG and thus were entitled to enroll into GK [16]. In November 2016, about 10,000 people were active members of GK. A feature of the Kinzig valley region is its rural character with towns of up to 11,000 inhabitants. The distance to the closest university hospital, located in Freiburg, is at least 45 min by car. GK pursues the ‘Triple Aim’: to improve the health of the population, to improve the individual’s experience of care, and at the same time, to lower the healthcare costs per inhabitant [17]. To reach the first two components of the ‘Triple Aim’, the GK management attempts—on the one hand—to strengthen and rationalize the cooperation between the various healthcare providers across professions and sectors and—on the other hand—to implement special prevention and health promotion programs. One very important field of action is to empower and activate patients and help them improve their health literacy [13,16].

In this article, we address the following explorative research questions:

1. What is the proportion of respondents with sufficient (compared to problematic and inadequate) health literacy in our GK sample?
2. Does health literacy correlate with quality of life and subjective health status in our sample? Do sociodemographic variables like age, gender, and duration of school education act as confounders in this relationship?

## 2. Methods

### 2.1. Data Collection

In a cross-sectional survey, 3218 randomly selected GK members were asked in 2017 about their satisfaction with their trusted doctors, with the integrated healthcare system as a whole and about patient-reported outcomes such as their subjective health status, health-related quality of life, and patient enablement on the basis of a self-administered standardized questionnaire. The questionnaire contained 75 items. This cross-sectional survey was part of a trend study [16] in which the changes in GK members’ subjective health status, perceptions etc. were to be monitored. From those 3218 GK members a subgroup of 597 persons was randomly selected for the present study. They received an enlarged questionnaire with additional questions on health literacy and general self-efficacy; the enlarged questionnaire version contained a total of 101 items. Three weeks after the questionnaire had been sent to the selected GK members, they received a written reminder. The study was positively reviewed by the Ethics Committee of the University of Freiburg (Az. 294/12_140826).

In the cover letter to the questionnaire, survey participants were informed about the voluntary nature of their participation and about data protection regulations. By returning the completed questionnaire, respondents gave their consent to participate in the study. There were no (financial or non-financial) incentives for respondents to take part in the study.

#### Instruments

The standardized questionnaire included the EQ-5D (three-level version, response options: no, some, extreme problems/unable to) to assess health-related quality of life and the respective visual analogue scale (EQ-VAS, three-level version) to assess subjective health status [18], the European Health Literacy Survey Questionnaire with 16 items (HLS-EU-Q16) [19,20], the Generalized Self-Efficacy Scale (GSE) [21], and the ‘Patient Enablement Scale’ with 13 items (PEN-13) [22].

The EQ-5D [18] is a standardized instrument to measure health-related quality of life and subjective health status for clinical and economic appraisal [18]. Based on five items, it provides both a single index for health-related quality of life (EQ-5D index) and a self-report vertical visual analogue scale (EQ-VAS) (‘0’ worst imaginable health status to ‘100’ best imaginable health status) [23]. The HLS-EU-Q16 is a comprehensive measurement of health literacy with 16 items derived from HLS-EU-Q47 [24]; both versions capture a broad public health perspective [25]. The HLS-EU-Q16 represents the three domains healthcare, disease prevention, and health promotion [26]. This short version comprises the four components: accessing, understanding, appraising, and applying health-related information, but the focus of this instrument is on finding and understanding information [26]. The GSE scale captures general self-efficacy unidimensionally and is an internationally widespread, standardized instrument with 10 items [21,27]. The PEN-13 is a newly developed generic German measurement instrument to capture patient enablement by 13 items and has two dimensions [22].

### 2.2. Study Participants

The study participants were registered GK members (see Section 1) and were insured by the statutory health insurers AOK BW or SVLFG. Among GK members, AOK BW insureds outnumber SVFLG insureds by far: in November 2016 there were more than 20 times as many AOK BW insureds than SVLFG insureds among GK members [28]. GK members remain free to choose their doctors and other service providers, and they may resign from the GK system at the end of a quarter. Registered GK members can use certain local (health) services free of charge or at a lower price than non-members. For example, members can attend lectures and presentations on health-related topics for free, participate in courses on exercise and nutrition, or train in a GK gym at low cost. There are no costs or financial incentives for becoming a member.

The members of the GK system are of all ages and in varying health conditions. Women, the elderly, and people with multi-morbidity and (multiple) chronic diseases have been—at least during the start-up phase of the GK system founded in 2005—clearly over-represented among enrolled GK members in comparison to all AOK BW insureds in the region [13]. However, there are also GK members who currently do not receive any medical treatment and may not have visited their general practitioner (or other health providers) for a longer time. According to the 2015 GK member survey, 20% of the responding members consulted their trusted doctor no more than once in the past year [29].

### 2.3. Statistical Analysis

The sociodemographic characteristics as well as the health-related scales EQ-5D index, EQ-VAS, HLS-EU-Q16, GSE, and PEN-13 were first analyzed descriptively. If a participant had more than two missing HLS-EU-Q16 items (the answer category “do not know” was also interpreted as a missing value [20]), a HLS-EU-Q16 score was not calculated. This procedure is the standard one and corresponds to that of Röthlin et al. [20] and to that of a representative German study [30]. The definition of “sufficient health literacy” (score values 13–16 in HLS-EU-Q16) as well as the rule to define study participants with a valid HLS-EU-Q16 score (<2 missing answers) were derived from Röthlin et al. [20].

We calculated the quality of life score (EQ-5D index) [18], the health literacy score (HLS-EU-Q16) [20], as well as the proportion of people with sufficient health literacy (research question 1) with the respective confidence interval. Subsequently we computed mean differences between groups (t-test) and bivariate and partial correlation coefficients between HLS-EU-Q16 and EQ-5D index or EQ-VAS, respectively; sociodemographic variables were determined to explore key features of health literacy in this sample (research question 2). A *p*-value of <0.05 was taken to indicate statistical significance. All analyses were performed with SPSS version 25 (IBM Corp., Armonk, NY, USA).

We calculated partial correlations between HLS-EU-Q16 on the one hand and EQ-5D index and EQ-VAS on the other, with the control variables age, gender, and duration of school education (research question 2). In order to make the results clearer, more comparable, and to have a metrically scaled variable, we worked with ‘years of school education’ in addition to school-leaving certificates. As survey participants were asked in the questionnaire only about their highest school-leaving certificate (‘höchster Schulabschluss’ in German), we transformed respondents’ original answers in the following way: participants with ‘Hauptschule’ (lowest secondary school certificate in Germany) as highest certificate were assigned 9 years of school education; participants with ‘Realschule’ (intermediate maturity) were assigned 10 years of school education; respondents with ‘Fachhochschulreife’ (advanced technical college certificate) were assigned 12 years, and participants with ‘Abitur’ (a-level) were assigned 13 years of school education. Respondents without any school-leaving certificate were assigned 8 years of school education due to compulsory schooling in Germany.

## 3. Results

### 3.1. Sociodemographic and Health-Related Characteristics

Of the 597 questionnaires sent out, 180 were completed and returned (response rate 30.2%). There were no missing data for age and gender; the 180 study participants were on average 63.7 (standard deviation (SD) = 16.1) years old; the proportion of women was 56.1%. More than half of the participants indicated to suffer from chronic disease; the proportion of currently employed people amounted to 35.6% (see Table 1 for the sociodemographic characteristics of the study participants and Table 2 for the descriptive results of the health-related scales). A total of 176 study participants answered the question whether they had ever taken part in an activity offered by GK. Of these, 31.8% (*n* = 56) indicated they had already participated, while 68.2% (*n* = 120) reported they had never participated in any GK activity before.

### 3.2. Relative Frequency of HLS-EU-Q16 Levels

An HLS-EU-Q16 score could be calculated in 126 subjects; it averaged 12.2 points (SD = 4.2), ranging between 0 and 16. According to the manual, three different levels of health literacy can be defined [20]. The distribution of these levels can be seen from Table 3 (results of research question 1). Sufficient health literacy resulted in 61.9% of respondents, whereas 19.8% had problematic health literacy and 18.3% had inadequate health literacy.

The proportion of respondents for whom no HLS-EU-Q16 score could be calculated was 30% (*n* = 54 with more than two missing HLS-EU-Q16 items). We explored whether participants with and without a valid HLS-EU-Q16 score differed in age, gender, education, employment status, and health outcomes. As can be seen in detail from Table S1 (metrically scaled variables) and Table S2 (nominally scaled variables)—see Supplementary Material, respondents with vs. without valid HLS-EU-Q16 scores did not differ as to age, years of school education, employment status, their GSE and PEN-13 scores, but as to their EQ-5D index and EQ-VAS score. Adjusted for age, gender, and duration of school education, participants without a valid health literacy score showed a better health-related quality of life, measured by EQ-5D index (F (4. 163) = 6.04; *p* < 0.001, partial eta^2^ = 0.132), and a better current subjective health status, measured by EQ-VAS (F (4.159) = 9.76; *p* < 0.001, partial eta^2^ = 0.201), compared to participants for whom a valid score could be calculated.

### 3.3. Sociodemographic Variables and Health Literacy

There was no statistically significant difference between the mean health literacy score of female and male study participants (*t* (124) = −0.495, *p* = 0.621). There was also no significant difference in the mean health literacy score between chronically ill and non-chronically ill persons (*t* (121) = −0.066, *p* = 0.947). Furthermore, there was no significant difference between the mean values of employed and non-employed study participants (*t* (112) = 1.945, *p* = 0.054). Similarly, the study participants who had already participated in a GK activity and those who had not yet participated in any of the offered activities could not be distinguished in terms of their mean health literacy scores (t (103) = 0.307, *p* = 0.760).

The bivariate Pearson correlation coefficient between health literacy and age was not significantly different from ‘0’. We found however a significant relationship between health literacy and duration of school education (as well as highest school-leaving certificate) in our sample: in both cases, the relationship was positive, i.e., the higher the duration of school education (or school-leaving certificate), the higher the HLS-EU-Q16 score value. Table 4 shows the bivariate correlations with the HLS-EU-Q16 score.

### 3.4. Health Literacy and Health-Related Quality of Life and Subjective Health: Bivariate and Partial Correlations

The bivariate correlation coefficient between HLS-EU-Q16 and EQ-5D index was r = 0.41, between HLS-EU-Q16 and EQ-VAS the correlation coefficient amounted to r = 0.40. Controlled by age, gender, and duration of school education, partial correlations of r = 0.36 (EQ-5D index) and r = 0.37 (EQ-VAS) resulted. All correlations were significant (*p* < 0.001) (Table 5).

## 4. Discussion

This article is the first examining the question of how health literacy was distributed in a sample of registered members of a regional integrated healthcare system in Germany whose strategic goal is the empowerment and activation of its members with regard to health issues. Our results showed that the level of health literacy among our survey participants is higher compared to other nationwide studies using the HLS-EU-Q16. In our sample, which consists mainly of insureds by AOK BW, 61.9% had sufficient health literacy (HLS-EU-Q16 score values 13–16). Our category “sufficient health literacy” includes those who reported either sufficient or excellent health literacy. In a representative study of only AOK insureds [31], a lower level of health literacy resulted than in a nationwide German survey covering insureds of the statutory and private health insurance funds [30]: in the AOK study, the analogous proportion of participants with sufficient health literacy was 40.4 %, whereas in the nationwide survey 55.8% of respondents indicated sufficient health literacy.

In light of these other surveys—and keeping in mind that we had a sample mainly consisting of AOK BW insureds with a low level of education on average—the comparably high proportion of respondents with sufficient health literacy could indicate that the emphasis on prevention, health promotion, and empowerment of the GK management had a positive effect on the level of health literacy of GK members.

In addition to this result, we examined the correlation between HLS-EU-Q16 and sociodemographic variables in our sample. We could not find a significant association between health literacy and age, gender, the presence of a chronic disease or participation in GK activities; we could however state a significant correlation between health literacy and educational status depicted by highest school leaving certificate (r_s_ = 0.34, *p* < 0.001) or duration of school education (r = 0.30, *p* < 0.001). This significant relationship between education and health literacy is usually observed [32] and was also found in Germany (e.g., [4,30,31]).

In contrast, the findings on the relationship between health literacy and age and also between health literacy and gender are inconclusive. Likewise, Jordan and Hoebel [30], for example, found no significant associations between age and gender on the one side and health literacy on the other; this is in line with the results of the European health literacy survey in Germany [4]. In the above-mentioned AOK survey, however, women showed a higher level of health literacy than men, and the average level of health literacy tended to increase with age [31]. In our sample, respondents are quite homogeneous in age; it is possible that other studies with a wider age range provide more insights into the relationship between age and health literacy.

To check whether the association between health literacy and quality of life and subjective health status is similar to what has been found in other studies (e.g., [33,34,35,36]), we first computed bivariate correlations between the respective variables and, in a second step, partial correlations with which we eliminated the influence of the control variables age, gender, and duration of school education. The results to our second research question can be summarized thus: the moderate bivariate correlation (r = 0.35) between health literacy and quality of life found by Zheng et al. [10] in their meta-analysis could largely be confirmed in our sample: between health literacy and the two health outcomes (health-related quality of life and subjective health status), bivariate correlations of 0.41 (EQ-5D index) and 0.40 (EQ-VAS) resulted. Remarkably, these correlation coefficients hardly decreased when the control variables were considered by computing partial correlations between health literacy and the two health outcomes—in this case partial correlations of 0.36 (EQ-5D index) and 0.37 (EQ-VAS) resulted. This indicates that the control variables considered here hardly confounded the relationship between health literacy and the two health outcomes.

With our sample, we have addressed a very specific population: on the one hand by the region in which the participants lived and on the other hand by the addressed group of insureds who had enrolled into a regional integrated healthcare system. GK strives to promote health-related empowerment and activation of its members and has various special offers for them. The objectives of the GK system have always been to promote members’ self-management capabilities (see Section 1). These GK system characteristics could have contributed to an above-average level of health literacy in our study participants—despite the fact that the educational level of our sample population seems to be rather low (Table 1), even when we consider the comparably high age of the sample and the rural character of the study region.

In our postal survey, remarkably, there was a high proportion of respondents (30%; *n* = 54) with more than two missing HLS-EU-Q16 items; for these respondents no health literacy score could be calculated. A study in the Netherlands had a similar quota for non-calculable HLS-EU-Q16 scores; there were 33% of the study participants without a valid score [37]. In the data collection of the European Health Literacy Survey, participants were—contrary to our study—interviewed personally with either computer or paper assistance [4,5,33]. Therefore, the problem of missing values, as it was the case with the short Q16 version in our postal survey, occurred much less frequently. When we compared the characteristics of study participants with and without a valid health literacy score, interestingly, participants without a valid score reported a significantly higher subjective health status. The difference between participants with and without a valid health literacy score regarding health-related quality of life and current health status could possibly be explained by the fact that people with a better health have less experience in searching and accessing health-related (and illness-related) information and for this reason have not responded to some HLS-EU-Q16 items.

## 5. Limitations

When we defined the values of the variable ‘duration of school education’ based on the originally surveyed variable ‘highest school-leaving certificate’ we did not take into account the fact that the years of schooling up to graduation and the years of compulsory schooling were different for people of different age groups. In addition, ‘duration of school education’ is only a partial measure of one’s educational status, for not only school education influences the social and socioeconomic status of the individuals interviewed, but also their further qualifications which people acquire in the course of their professional career. This shortcoming should be removed in future studies; for example, the application of an international qualifications framework (e.g., the International Standard Classification of Education—ISCED) could be more useful than just surveying the levels (or duration) of school education.

All in all, we must consider the relatively small sample size and a potential response bias in our study. The response rate of 30% can be regarded as moderate or even low, although it is within the range of what can be expected from postal surveys in which there are no special incentives for potential respondents to take part [38]. One should consider that the length of the entire questionnaire, covering 10 pages, can be a barrier for many potential respondents—in particular for those who are not accustomed to reading abstract texts. This leads us to a severe limitation of our study: it seems at least possible (if not probable) that those GK members whose general literacy is above-average or who have an increased interest in personal health and other health issues took part in the survey to a larger degree than those GK members with a low general literacy or less interest in those issues; for this reason the level of health literacy in the GK member population might appear too high on the basis of our data. It seems, however, difficult to assess the impact of such a potential selection bias on the comparison with the prevalence of ‘sufficient health literacy’ because the authors of the nationwide survey also refer to a potential selection bias in their study [30].

Our postal survey suggests that the HLS-EU-Q16 is more suitable for personal interviews, i.e., when an interviewer can collect all items and inquire the reasons why some statements may—or may not—apply to participants. Thus, when planning future studies with study participants using self-administered paper-and-pencil questionnaires, the use of the HLS-EU-Q16 questionnaire should be critically reconsidered.

## 6. Conclusions

We surveyed a comparably homogeneous group of insured persons enrolled in an integrated healthcare system located in a rural and small-town region in the Black Forest in southwestern Germany. In this sample we were able to demonstrate moderate correlations between health literacy on the one hand and quality of life and subjective health status on the other, confirming the findings of Zheng et al. [10]. Our study participants indicated a higher health literacy in comparison to nationwide German surveys based on the same outcome measure [30,31]. It can be assumed that the healthcare situation in the region, shaped by the integrated healthcare system with its emphasis on prevention, health promotion, as well as patient empowerment and activation, contributed to this result; it remains unclear, however, how strongly a possible selection bias interfered. However, to check—with sufficient evidence—whether the GK system leads to (i.e., is the cause of) a higher level of health literacy among GK members in comparison to the general population, a more rigorous study design (controlled study) with a larger sample size would have to be implemented in a further study.

## Figures and Tables

**Table 1:** Sociodemographic characteristics of the study participants.

Sociodemographic Characteristics		*n* = 180	
		*n*	%
Gender			
Male		79	43.9
Female		101	56.1
Age (Years)			
Mean (Standard Deviation)	63.7 (16.1)		
Median	66.0		
Range	21–95		
Chronic Disease			
Yes		104	57.8
No		56	31.1
Do not know		12	6.7
Missing		8	4.4
Education level			
No school leaving certificate		1	0.6
Secondary school certificate		118	65.6
Intermediate maturity		44	24.4
Advanced technical college certificate		6	3.3
Abitur (a-level)		6	3.3
Missing		5	2.8
Average years of schooling	9.5		
Years of schooling, median	9.0		
Employment status			
Currently employed		64	35.6
Currently not employed		100	55.6
Missing		16	8.9

**Table 2:** Description of the scores for health-related quality of life (EQ-5D index), health status (EQ-VAS), health literacy (HLS-EU-Q16), general self-efficacy (GSE), and patient enablement (PEN-13).

Scale	*n*	Mean (SD)	Range in Sample	Theoretical Range
EQ-5D index	167	0.83 (0.20)	0.09–1	−0.205–1
EQ-VAS	161	66.61 (21.20)	5–100	0–100
HLS-EU-Q16	126	12.19 (4.18)	0–16	0–16
GSE scale	162	29.00 (5.58)	10–40	10–40
PEN-13	173	51.57 (8.56)	22–65	13–65

**Table 3:** Relative frequency of HLS-EU-Q16 levels (defined according to [20]).

Levels of Health Literacy (*n* = 126)	*n*	%	95% Confidence Interval *
Sufficient health literacy (score values 13–16)	78	61.9	52.8–70.4
Problematic health literacy (score values 9–12)	25	19.8	13.3–27.9
Inadequate health literacy (score values 0–8)	23	18.3	11.9–26.1

* Method used for determining the 95% confidence interval: Clopper–Pearson.

**Table 4:** Bivariate Pearson’s (r) or Spearman’s (r_s_) correlation coefficients with HLS-EU-Q16.

Parameter	Age	Highest School Leaving Certificate	Duration of School Education
r (or r_s_ )	r = −0.133	r_s_ = 0.339	r = 0.299
*p* (two-tailed)	0.138	<0.001	<0.001
*n*	126	124	124

**Table 5:** Bivariate and partial correlations between HLS-EU-Q16 and quality of life (EQ-5D) and health status (EQ-VAS).

Scale	Bivariate Correlations with HLS-EU-Q16	Partial Correlations * with HLS-EU-Q16
EQ-5D index	r = 0.41 (*p* < 0.001), *n* = 118	r_partial_ = 0.36 (*p* < 0.001), *n* = 117
EQ-VAS	r = 0.40 (*p* < 0.001), *n* = 114	r_partial_ = 0.37 (*p* < 0.001), *n* = 113

* The control variables are age, gender, and duration of school education.

## Supplementary Materials

The following are available online at https://www.mdpi.com/1660-4601/17/5/1683/s1, Table S1: Sociodemographic and health outcome differences between participants with vs. without valid HLS-EU-Q16 score., Table S2: Sociodemographic and health outcome differences between participants with vs. without valid HLS-EU-Q16 score.
