# Supplementary material for: The Relationship between Health Literacy, Quality of Life, and Subjective Health: Results of a Cross-Sectional Study in a Rural Region in Germany
Source: Int J Environ Res Public Health. 2020 Mar 5;17(5):1683. doi: 10.3390/ijerph17051683 (PMC7084276; doi:10.3390/ijerph17051683)
Supplement: Supplementary file 1 [file ijerph-17-01683-s001.pdf]

Supplementary Materials: Table S1 and Table S2

Table S1. Sociodemographic and health outcome differences between participants with vs. without valid HLS-EU-Q16 score.

Parameter	HLS-EU-Q16 score	*n*	Mean	SD	t-Test	ANOVA *
Age	Valid	126	63.99	16.22	t(178) = −0.434; $p = 0.665$.	-
	Not valid	54	62.85	16.05		
Years of school education	Valid	124	9.51	0.95	t(173) = −0.489; $p = 0.625$	-
	Not valid	51	9.43	0.92		
EQ-5D index	Valid	118	0.82	0.22	t(165) = 1.113; $p = 0.267$	F(4) = 6.044; $p < 0.001$; partial $eta^2 = 0.132$
	Not valid	49	0.86	0.15		
EQ-VAS	Valid	114	64.90	21.82	t(159) = 1.975; $p = 0.050$	F(4) = 9.758; $p < 0.001$; partial $eta^2 = 0.201$
	Not valid	47	71.70	18.90		
GSE score	Valid	116	28.64	5.65	t(160) = 1.337; $p = 0.183$	F(4) = 0.533; $p = 0.712$; partial $eta^2 = 0.014$
	Not valid	46	29.93	5.35		
PEN-13	Valid	125	51.57	8.58	t(171) = 0.020; $p = 0.984$	F(4) = 1.943; $p = 0.106$; partial $eta^2 = 0.045$
	Not valid	48	51.59	8.58		

* The control variables were age, gender and duration of school education.

Table S2. Sociodemographic and health outcome differences between participants with vs. without valid HLS-EU-Q16 score.

HLS-EU-Q16 Score	Gender			Currently Employed		
	**Female**	**Male**	**Chi^2 Test**	**Yes**	**No**	**Chi^2 Test**
Valid	$n = 71$ (56.3%)	$n = 55$ (43.7%)	$chi^2 = 0.010$; $p = 1.000$ (exact sig. 2-sided)	$n = 46$ (40.4%)	$n = 68$ (59.6%)	$chi^2 = 0.276$; $p = 0.728$ (exact sig. 2-sided)
Not valid	$n = 30$ (55.7%)	$n = 24$ (44.4%)		$n = 18$ (36.0%)	$n = 32$ (64.0%)	
